# Edge-Machine-Learning-Assisted Robust Magnetometer Based on Randomly Oriented NV-Ensembles in Diamond

**DOI:** 10.3390/s23031119

**Published:** 2023-01-18

**Authors:** Jonas Homrighausen, Ludwig Horsthemke, Jens Pogorzelski, Sarah Trinschek, Peter Glösekötter, Markus Gregor

**Affiliations:** 1 Department of Engineering Physics, Münster University of Applied Sciences, Stegerwaldstraße 39, 48565 Steinfurt, Germany; 2 Department of Electrical Engineering and Computer Science, Münster University of Applied Sciences, Stegerwaldstraße 39, 48565 Steinfurt, Germany

**Keywords:** NV center in diamond, quantum sensing, magnetometry, edge machine learning, microcontroller, optically detected magnetic resonance, neural networks

## Abstract

Quantum magnetometry based on optically detected magnetic resonance (ODMR) of nitrogen vacancy centers in nano- or micro-diamonds is a promising technology for precise magnetic-field sensors. Here, we propose a new, low-cost and stand-alone sensor setup that employs machine learning on an embedded device, so-called edge machine learning. We train an artificial neural network with data acquired from a continuous-wave ODMR setup and subsequently use this pre-trained network on the sensor device to deduce the magnitude of the magnetic field from recorded ODMR spectra. In our proposed sensor setup, a low-cost and low-power ESP32 microcontroller development board is employed to control data recording and perform inference of the network. In a proof-of-concept study, we show that the setup is capable of measuring magnetic fields with high precision and has the potential to enable robust and accessible sensor applications with a wide measuring range.

## 1. Introduction

In the past few years, negatively charged nitrogen vacancy (NV) centers in diamond have emerged in the field of quantum-based high-sensitivity magnetic sensing. The sensor material offers high sensitivity reaching below nT/Hz${}^{-1/2}$ [1,2,3], outstanding spatial resolutions down to atom size [4,5,6] and a high dynamic range [2], while operating under room temperature and ambient conditions in a solid-state package. Furthermore, the directional sensing characteristic of individual NV electron spins enables ensembles of NV centers in monocrystalline diamonds to inherently deliver triaxial information used for vectormagnetometry [2,7,8,9,10]. These properties are attracting the increasing interest of scientists and companies, with the focus of research shifting more and more from fundamental physics to applied sciences and technical implementation [11,12]. In comparison to the widely used bulk diamond slabs [1,2,8,9,10,13], micro-diamonds offer low production costs, high availability and scalability, further enhancing the appeal of this sensor material in an industrial application context.

**Figure 1:**
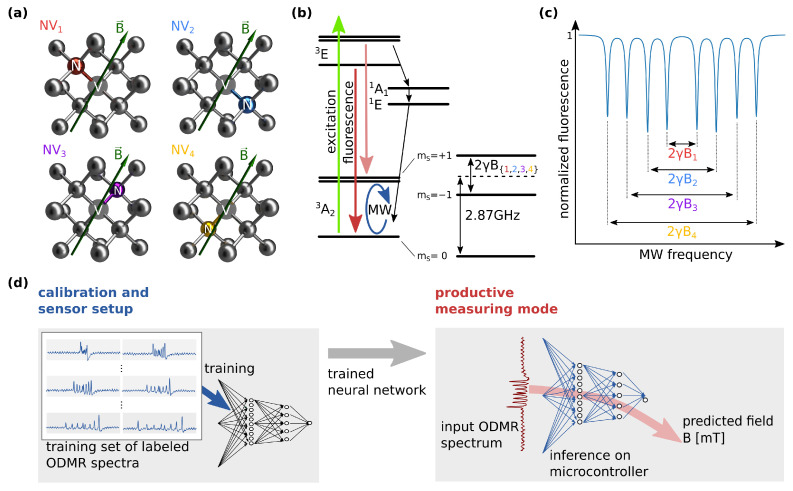
(**a**) Four orientations of the NV center in the diamond crystal structure. (**b**) Simplified energy level system of the NV center electron spin. Decay from the triplet excited state ${}^{3}E$ into the ground state ${}^{3}A_{2}$ leads to a fluorescence in the visible spectrum with a zero-phonon line at 637nm [14]. Decay from the $m_{S}=\pm 1$ excited state increases the probability of a transition via a singlet state with fluorescence in the non-visible spectrum. (**c**) Schematic ODMR spectrum showing the fluorescence intensity as a function of MW frequency as indicated in (**b**). Different projected magnetic fields $B_{i}$ lead to different Zeeman shifts and thereby eight distinct dips in fluorescence. (**d**) Schematic of the machine learning approach. A dataset of labeled ODMR spectra is used to train a neural network on a workstation. The trained neural network with fixed weights is then transferred to the embedded device, where the magnetic field is predicted from a single recorded ODMR spectrum by network inference in productive measuring mode.

The NV center is a point defect in the carbon lattice of diamond, in which a substitutional nitrogen atom (N) is accompanied by an adjacent vacancy (V). Figure 1a shows the four orientations in which NV centers can be present in the diamond crystal structure, denoted here as ${\mathrm{NV}}_{i}$, where i∈{1,2,3,4}. The NV center introduces additional energy levels in the band gap of the diamond (Figure 1b). In the triplet ground state ${}^{3}A_{2}$, the $m_{S}=\pm 1$ sublevels are shifted from the $m_{S}=0$ sublevel by D=2.87GHz. Due to the Zeeman effect, the $m_{S}=\pm 1$ levels are shifted additionally in the presence of a magnetic field by $\Delta f=2\gamma B_{i}$, where γ=28MHz/mT is the gyromagnetic ratio and $B_{i}$ are the components of the projected magnetic field parallel to the symmetry axes of the NV centers. We observe the Zeeman shifts by detecting dips in fluorescence intensity, which are caused by a higher probability of a decay via the singlet states ${}^{1}A_{1}$ and ${}^{1}E$ (Figure 1b) if the NV center is spin-polarized via resonant microwave excitation. Fluorescence is measured in an optically detected magnetic resonance (ODMR) experimental setup. Note that in NV ensembles, NV centers in all four shown directions are present. The projections of a magnetic field onto the four possible orientations of the NV centers lead to different effective magnetic fields parallel to their symmetry axes. In Figure 1c these are denoted as $B_{i}$, respective to the ${\mathrm{NV}}_{i}$ orientations. They result in different Zeeman shifts and thereby eight distinct dips in fluorescence during a frequency sweep of the microwave (MW) excitation [8]. Since the positions of these resonances in the spectrum are determined by the respective fields $B_{i}$, the spectrum depends on the orientation of the crystal with respect to the magnetic field vector [2,9].

Physical models and theories that describe the interaction of the NV electron spin with magnetic, electrical and strain fields are well known [7,15,16]. However, to apply these models, exact information about the crystal orientation is essential [2,10,17]. When using randomly oriented micro-diamonds in a magnetometry context, the precise characterization of the crystal orientation can be challenging [9,18], making it difficult to apply these physical models. Furthermore, the interaction of crystal strain fields [13,15,16] and off-axis magnetic fields in the higher field regime [15,19] bring additional parameters into the physical model along with rising complexity. These challenges limit the accuracy and applicability of approaches employing physical models to deduce magnetic fields from measured ODMR spectra. To overcome this problem, machine learning techniques without application of a physical model have recently been proposed as an alternative approach in ODMR quantum magnetometry. In [20], Tsukamoto et al. applied Gaussian process regression for randomly oriented NV ensembles in nano-diamonds, reaching high accuracy of 1.8μT for fields of 1000–1500μT. In the context of a single-spin readout at room temperature, where the signal is typically noisy and thus hard to process, neural networks have recently been proposed to extract the position of resonance peaks in ODMR spectra [21]. In addition, machine learning has been shown to drastically speed up the measuring process in ODMR magnetometry by using Bayesian experiment design instead of a conventional frequency-swept measurement [22]. In addition, it has been employed to systematically optimize measuring parameters such as laser and microwave power in ODMR experiments [23].

Here, we propose employing artificial neural networks to implement a scalar magnetic-field sensor based on randomly oriented NV ensembles in diamond without the need for the characterization of the crystal orientation and the use of physical models. The machine learning approach is sketched in Figure 1d. A neural network is first trained on a workstation with data acquired from a continuous wave (CW) ODMR setup. The training dataset consists of measured ODMR spectra as well as the corresponding magnetic field. Then, the trained model is converted to a format optimized for usage on microcontrollers and deployed to an embedded device. Inference of the network is performed locally during measurements in the productive mode of the sensor device. Thereby, the magnetic field is predicted by the neural network from newly recorded ODMR spectra. Note that the neural network is adapting to the specific shape of the recorded spectra with the particular sensor device during the training process. This in particular also applies to systematic measurement errors and deviations from the theoretically expected signal. Therefore, the presented method reduces the requirements placed on measurement quality as compared to approaches that determine the magnetic field by fitting a physical model to the data.

The concept of employing machine learning techniques directly on embedded end devices to perform on-device sensor data analytics, so-called edge machine learning, has recently gained a lot of attention (see [24,25,26] for reviews). It enables the development of intelligent sensor systems with extremely low power consumption that do not need to exchange data with a cloud server (where machine learning algorithms would traditionally be performed). Thereby, edge machine learning not only allows for sensor operation in harsh environments without network connection, but also reduces the latency in a sensor application. Naturally, implementing machine learning techniques on resource- and performance-constrained embedded devices is challenging but has been greatly facilitated in the last couple of years by the development of edge computing frameworks, such as TinyML [27,28,29]. In this work we show that in the context of quantum magnetometry, edge machine learning has great potential for designing robust, accessible low-cost sensor devices with a high dynamic range that do not rely on fitting experimental data with physical models. The resolution of the sensor is mostly determined by the step size of the ODMR microwave sweep and the neural network complexity. It can therefore be chosen according to the requirements of the sensing application via a trade-off with the required amount of training data and the temporal resolution in the measuring mode.

In the following, we first introduce the experimental setup required to record ODMR spectra as training data, as well as in the productive mode. Next, we describe our edge machine-learning approach, including the choice of network architecture, the training process and the deployment to an embedded device. Then, the performance of the sensor system is evaluated and its advantages as well as its limitations are discussed. Lastly, we give an outlook for the application of edge machine learning for ODMR.

## 2. Materials and Methods

### 2.1. Experimental Setup

**Figure 2:**
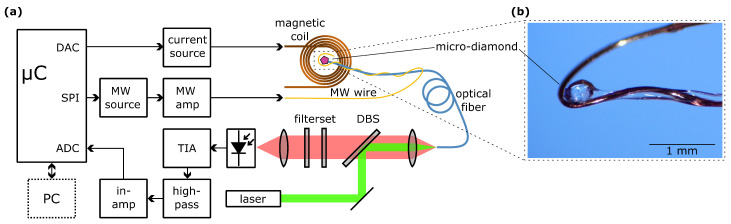
(**a**) Schematic of the ODMR experimental setup. By applying a microwave frequency sweep to the micro-diamond with NV centers, ODMR spectra are acquired using the built-in ADC of the microcontroller. (**b**) The fiber-coupled NV micro-diamond is fixed onto the tip of the optical multimode fiber with UV-cured adhesive. A 100μm wire is wrapped around the fiber and brought into close proximity with the micro-diamond for MW excitation.

The experimental setup used in this work is a basic CW ODMR setup as shown in Figure 2a. The main feature of this setup is the simultaneous excitation of the NV centers with laser light at 532nm, as well as microwave radiation at around 2.87GHz. If the microwave frequency is tuned to resonance with one of the $m_{S}=\pm 1$ sublevels, a decrease in fluorescence intensity can be observed due to increased relaxation via the (dark) singlet states shown in Figure 1b. A full ODMR spectrum can be acquired by sweeping the MW frequency.

In the following, we briefly describe the micro-diamond sample used as the sensing material, the optical setup used for excitation with laser light and detection of the fluorescence light, as well as the electronic setup used to control the microwave radiation, measure the fluorescence signal and control the magnitude of the magnetic field.

#### 2.1.1. Diamond Sample

The diamond sample used in this work is a micro-diamond with a size of approximately 150μm distributed by Adámas Nanotechnologies, Raleigh, NC, USA (MDNV150um Hi50mg). These diamonds have an NV concentration of 2.5–3ppm [30], which results in a very bright fluorescence that can easily be detected using commonly available photodiodes. In our measurements, we observed a considerable amount of crystal strain that caused a shift in the ODMR resonances even at zero magnetic field. As shown in Figure 2b, the micro-diamond is placed onto the end surface of an optical multimode fiber with a numerical aperture of NA=0.22 and a core diameter of 50μm (Thorlabs FG050UGA), where it is held in place by an optical adhesive (Norland 63) that has been irradiated by UV radiation from an LED source for ten minutes. The diamond is partly trapped inside the adhesive in order to increase the collection efficiency of fluorescence.

#### 2.1.2. Optical Setup

We used a CW DPSS laser module with a wavelength of 532nm and a power of approximately 30mW for excitation of the NV ensemble that is optically coupled to the optical fiber. As sketched in Figure 2a, the laser beam is reflected off a dichroic beamsplitter (DBS) with a 550nm longpass characteristic (Thorlabs DMLP550) and focused into the fiber core with a microscope objective with a numerical aperture of NA =0.25. The NV fluorescence is collimated by the same microscope objective, passes through the DBS and a bandpass filterset with a cut-on wavelength of 550nm (Thorlabs FEL0550) and a cut-off wavelength of 750nm (Thorlabs FES0750). This bandpass is tailored to the expected fluorescence spectrum of NV centers in diamond at room temperature [14] in order to suppress leakage of laser radiation and unwanted fiber fluorescence and thus increase the signal-to-noise ratio of the setup. Measurement of fluorescence intensity and data acquisition is further described in Section 2.2.2.

#### 2.1.3. Electronic Measurement Setup

To generate and collect data, a low-power, low-cost ESP32 microcontroller DevKit V2 board (ESP32) was used. This widely used board was chosen mainly based upon its TinyML support, and has additional advantageous features for this task such as two built-in DACs, fairly highly resolving ADCs with 12 bit and a serial peripheral interface (SPI). The electronic setup combines several tasks. In order to measure a single ODMR spectrum for a particular magnetic field magnitude, the frequency of microwave radiation needs to be controlled. In parallel, the intensity of the fluorescence signal has to be recorded. To generate exemplary ODMR spectra for different magnetic fields as training sets, as well as to test the sensor, the magnitude of the magnetic field needs to be controlled.

For the generation of microwave radiation, a radio frequency signal is synthesized by means of a circuit board that is based on an Analog Devices ADF4351 wideband synthesizer with an integrated voltage-controlled oscillator (VCO) and frequency divider. The main advantages of this board are the serial peripheral interface for configuration by the embedded device, the built-in phase-locked loop (PLL) to lock the output frequency and a resulting high-frequency band ranging from 35MHz to 4.4GHz that can be controlled by the ESP32. In this work, a frequency sweep with a center frequency of 2.87GHz and a span of 800MHz was performed in 400 discrete steps. This frequency span was chosen based on the maximum magnetic field of 11.5mT; however, a higher dynamic range can easily be achieved by increasing the frequency span of the sweep. The synthesizer is set to its minimum output power and one of the differential outputs is used, resulting in an output power of −7dBm. The signal is then amplified by at least 18dB using a microwave amplifier (Mini-Circuits ZRL-3500+). For emission of microwave radiation, a 100μm diameter copper wire is used as a microwave antenna. As shown in Figure 2b, the MW wire is wound around the last ten centimeters of the optical fiber in a helix-like manner, and is then bent into a single loop around the diamond. It is simply connected to the inner conductor of a coaxial cable without termination.

The fluorescence intensity is measured with a photodiode (BPW34) and amplified via a custom-built transimpedance amplifier (TIA), analyzed in Appendix A.1. To digitize the signal using the built-in 12-bit analog-to-digital converter (ADC) of the ESP32, it is first inverted and amplified using the circuit described in Appendix A.2.

To control the magnitude of the magnetic field, the integrated DAC of the ESP generates 250 different analog values, which in turn set different current values in the range 0 to 1.2A via a high-power operational amplifier circuit, based on a Texas Instruments OPA541. The current is fed to a coil and generates an adjustable DC magnetic field of 0 to 11.5mT.

### 2.2. Edge Machine Learning for ODMR Spectrum Analysis

#### 2.2.1. Conceptional Approach

**Figure 3:**
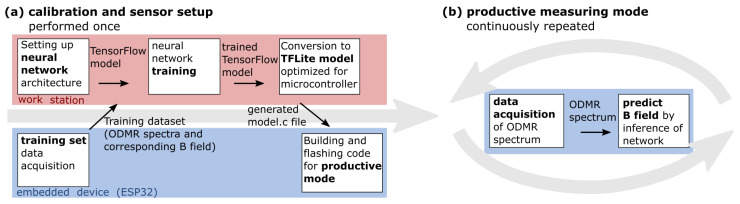
(**a**) Sequence of steps needed to calibrate and set up the ODMR sensor by deploying a trained edge ML model to the embedded device. (**b**) Productive measurement mode on the embedded device. One ODMR spectrum is acquired and used as input for the inference of the neural network to predict the B field. This two-step process is continuously repeated during measurement mode.

We propose using artificial neural networks in an edge-computing framework to deduce the magnetic field magnitude from recorded ODMR spectra. Since for each individual magnetic field sensor, the ODMR spectra obtained for a given magnetic field will differ due to the spatial orientation of the nano-diamond w.r.t. to the field, as well as other experimental conditions, a sequence of steps is necessary to calibrate and set up each individual sensor. This sequence is outlined in Figure 3a. First, a training set needs to be acquired, consisting of representative ODMR spectra obtained with the sensor as training data as well as the corresponding B field as the training label. These data are then used to train a neural network on a workstation (or, in later applications, possibly also a cloud server if desired). Note that in this work, two different neural networks were trained to explore the effect of the network architecture on the performance of the system. In a real industrial application, only a single neural network will be implemented and trained. The trained neural network is then deployed to the embedded device in the form of a C file as part of the code for productive mode. In this mode, the sensor is ready to perform new measurements by recording ODMR spectra and performing inference of the neural network on the device to predict the magnitude of the magnetic field.

In the following, we provide further details about the individual necessary steps in our method.

#### 2.2.2. Training Set Acquisition

In order to obtain a sufficient amount of labeled training data, data acquisition is carried out in an automated manner. In this, all control sequences are put out by the ESP32. To record representative spectra for different magnetic field magnitudes, the coil current is controlled by the internal DAC and the magnetic field magnitude is swept from 0.2mT to 11.5mT in 250 steps. For each magnetic field magnitude value, a single ODMR spectrum is recorded by incrementally increasing the microwave frequency via the SPI interface and acquiring 4 fluorescence intensity measurements for each frequency step with the internal ADC. These four measurements are averaged to suppress background noise. Due to 400 steps in a single microwave frequency sweep, the resulting digitized ODMR spectrum is represented in an array with 400 elements. This spectrum is transferred to the workstation PC via serial communication alongside the according label, namely the magnetic field magnitude at which the spectrum was recorded. For dataset acquisition, this control sequence can be run in an infinite loop until sufficient training data are acquired.

In a post-processing step performed on the workstation PC prior to training, the offset is removed from the ODMR spectra. To that end, the spectra are first shifted individually by a constant offset value determined by averaging 10 edge values on each side of the spectrum. Subsequently, they are normalized by the maximum value.

**Figure 4:**
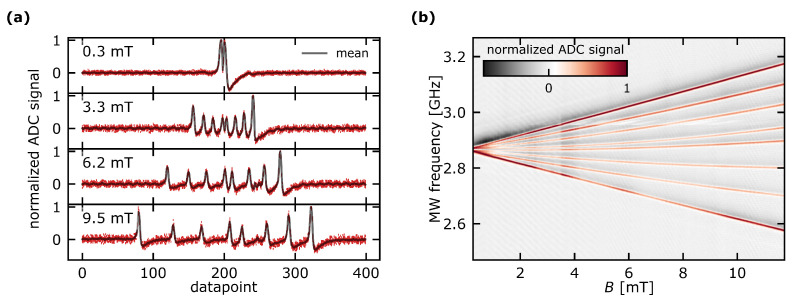
Exemplary visualization of the acquired data. (**a**) Ten exemplary training spectra at different magnitudes of the magnetic field. In a single ODMR spectrum, one datapoint is the mean, normalized value of four measured ADC values. Note that the dips in fluorescence are being inverted by the instrumentation amplifier. The undershoot at the right-hand side of the inverted dips can be attributed to the high-pass filter in the signal chain. (**b**) Dataset for a full sweep of magnetic magnitude.

Example datasets are depicted in Figure 4. Note that the dips in fluorescence are being inverted by an inverting buffer. Additionally, the high-pass filter in the signal chain introduces a small undershoot at the right-hand side of the inverted dips. Details regarding the inverting buffer are discussed in Appendix A.2.

The Zeeman splitting can be observed by the shifting of the fluorescence dips, respectively the resonant transition frequencies. In total, 8 dips can be observed representing the $m_{S}=+1$ and $m_{S}=-1$ spin transitions for all 4 NV axes present in NV ensembles. Here, the shift in the dips depends on the angle of the magnetic field vector and the NV symmetry axis. Specifically, the outermost fluorescence dips are attributed to the NV axis with the smallest angle to the magnetic field vector $\vec{B}$ and thus the highest axial magnetic field component $B_{z}$. Conversely, the innermost fluorescence dips indicate the smallest axial magnetic field component $B_{z}$ for the respective NV axis. Moreover, for NV axes with a small axial magnetic field component $B_{z}$, and a consequently large non-axial magnetic field component $B_{⊥}$, the resonance frequencies progress in a non-linear way as can be seen for the two inner fluorescence dips around 10mT in Figure 4b. From the theory of the NV ground state [15], this nonlinearity is expected to intensify with an increasing non-axial magnetic field component $B_{⊥}$.

Note that for small magnetic field magnitudes (B=0.3mT in Figure 4a), the 8 expected fluorescence dips are overlapping and superimposed, resulting in more pronounced fluorescence dips. The superposition, however, is not visible in the contrast to the data depicted due to the normalization of the spectra. In our experiments, the $m_{S}=\pm 1$ spin levels were split even at zero field due to the internal crystal strain [13].

#### 2.2.3. Neural Network Architecture

In this work, two widely used neural network architectures were compared to explore their performance for ODMR spectrum analysis: fully connected neural networks (FCNNs), which have conceptually the most simple network architecture, and convolutional neural networks (CNNs). The latter are particularly promising for the application in ODMR, since the magnitude of the magnetic field is encoded in clear structural features of the spectrum, namely the position of resonance dips. CNNs are capable of extracting and processing structural features in input data and are therefore typically employed to detect patterns and objects in image classification tasks. In magnetometry, the features detected by the CNNs are characteristics of the variation in the fluorescence signal with the microwave frequency.

**Figure 5:**
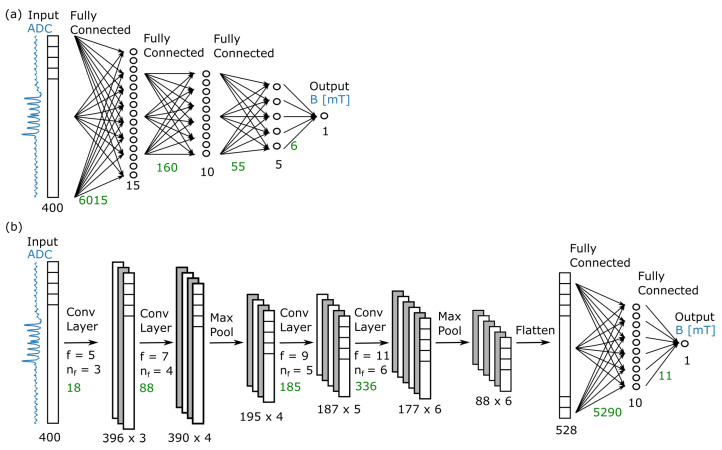
Employed neural network architectures of the fully connected neural network in (**a**) and the convolutional neural network in (**b**). The fully connected network has three hidden layers that decrease in size with the depth of the network. The convolutional neural network consists of a sequence of convolutional layers and max pooling layers, followed by two fully connected layers. The dimension of each layer is given in black; the number of trainable parameters per layer is given in green. For the CNN, *f* denotes the number of filters and $n_{f}$ denotes the kernel size applied in the respective convolution layer. The pool size of the max pool layers is 2.

Two different exemplary networks with a similar number of trainable parameters were implemented using the TensorFlow framework [31] and compared. Both networks have an input layer of size N=400, representing one ODMR spectrum with *N* sampling points, and a scalar output layer, representing the scalar magnetic field in mT. The architectures of the two presented networks were selected from a small hyper-parameter study in which characteristics such as the network depth and number of neurons and filters were varied. The employed fully connected network shown in Figure 5a consists of fully connected layers with a decreasing number of neurons using a ReLU activation function followed by an output layer with a linear activation function. In total, the network has 6236 trainable parameters. The employed convolutional network shown in Figure 5b is composed of a sequence of alternating convolution layers and max pooling layers, followed by a flattening layer, a fully connected layer and an output layer. It has 5928 trainable parameters. In addition, in the CNN, the layers have a ReLU activation function in all but the last linear layer. The technical implementation details, such as the kernel sizes and the number of filters in the convolution layers, are summarized in Figure 5b. The filter size of the convolution layers is small in the beginning to capture local features, i.e., changes in the fluorescence signal w.r.t. small variations in the microwave frequency, and increases with the depth of the network to capture broader structural features of the ODMR spectrum.

#### 2.2.4. Neural Network Training

Both neural networks were trained using the framework TensorFlow on a dataset consisting of the post-processed ODMR spectra recorded by the sensor device directly prior to training. The dataset was shuffled randomly and then split into 9960 training spectra and 2000 test spectra.

**Figure 6:**
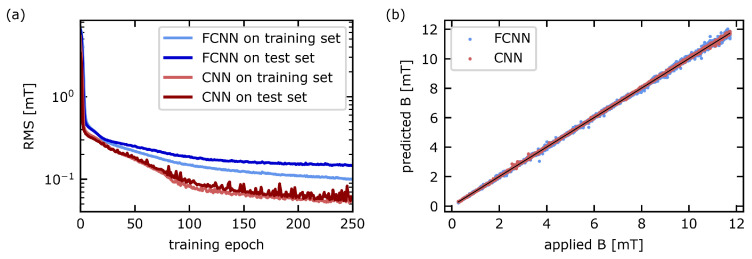
Comparison of fully connected (FCNN) and convolutional (CNN) network architectures for analysis of ODMR spectra. (**a**) shows the root-mean-square error (RMS) on the training and test set during the training process. (**b**) shows the predicted B field value depending on the “true” B field for 2500 randomly selected spectra from the test and training set.

The neural networks were trained on the training set for 250 epochs with a batch size of 128. The root-mean-square error (RMS) on the training set and the test set during training is shown in Figure 6a. The root-mean-square error of the FCNN on the test set (medium blue) initially rapidly decays and reaches a plateau after about 150 training epochs. The FCNN shows signs of over-fitting since the error on the training set continues to decrease with training. Conversely, the CNN does not show over-fitting and reaches a much smaller root-mean-square error. After 250 training epochs, the root-mean-square error on the test set averaged over the last 10 iterations of training is 139.2μT for the FCNN and 63.6μT for the CNN. For illustration, Figure 6b shows the prediction of the neural networks for 2500 randomly selected ODMR spectra from the recorded dataset against the known applied value of the B field. The predictions of both networks are distributed around the identity line (black). As expected from the training results described above, the CNN shows less scattering than the FCNN. For both networks, some outliers can be observed. These are caused by faulty ODMR spectra which, e.g., contain high noise, a tilted baseline or additional peaks due to irregularities of the laser.

#### 2.2.5. Deployment to the Embedded Device and Productive Mode

Since the performance of the CNN is much better than the performance of the FCNN for a comparable amount of trainable parameters as summarized in Table 1, we selected the CNN for deployment to the embedded device. Using the built-in TensorFlow converter, a TFlite model was generated from the trained neural network without quantization. The model was converted to a C byte array using standard tools. The size of the generated model depends on the model complexity. For the CNN, the model is 167KB. The byte array is then stored in the read-only program memory on the ESP32 device.

The C code for the productive mode was implemented using the TFLite C++ library [27]. Note that a very instructive introduction and examples for the usage of TensorFlow Lite for microcontrollers can be found in [28]. In productive mode, a single ODMR spectrum is first completely recorded using the routine previously employed for recording the training spectra (Section 2.2.2). Next, the spectrum data are post-processed in the same way as the training data on the workstation, i.e., the offset is shifted and the data are normalized by the maximum of the spectrum. Subsequently, the spectrum data are copied into the input tensor of the the TFLite model and inference is performed.

### 2.3. Sensor Performance

#### 2.3.1. Sensitivity and Accuracy

**Figure 7:**
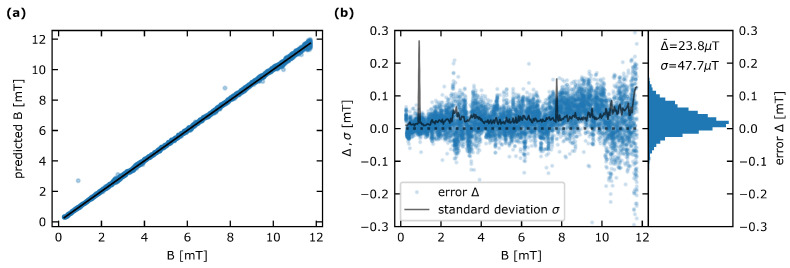
(**a**) Performance of the sensor device. In a total of 6250 measurements, the resulting RMS is 69.0μT. Single outliers can be attributed to random timing errors in the control sequence. (**b**) The error in the measurements $\Delta =B_{predicted}-B_{true}$ is trending towards a larger spread for increasing magnitudes of magnetic field. Accordingly, the standard deviation of the error σ shows a shift towards higher values. Right: The distribution of the error Δ for all 6250 measurements shows a mean value of $\bar{\Delta }=23.8\;\mu T$ and a standard deviation of σ=47.7μT.

To demonstrate the accuracy of the sensor, we performed multiple sweeps of the B-field magnitude while simultaneously logging the prediction of the neural network from the recorded ODMR spectrum. The data presented in the following stem from 25 of these B-field sweeps. With few exceptions, the datasets were recorded consecutively. In Figure 7, the results are shown. The predictions given by the CNN show a root-mean-square error of 69.0μT from the true magnetic field magnitude. The result agrees well with the RMS on the test set in the CNN training stage (Section 2.2.4).

The error Δ in the measurement, namely the difference in the predicted magnetic field magnitude and the true magnetic field magnitude, shown in Figure 7b, indicates a higher deviation for larger magnitudes of magnetic field. This trend is also evident in the course of the standard deviation of the error σ for rising magnetic field magnitudes (black line in Figure 7b). Note that the two peaks in the curve at around 1mT and 8mT stem from single outlying datapoints in the entirety of measurements. The standard deviation shows abruptly rising behavior for measurements close to the upper training limit in magnetic field magnitude, above 11.3mT in this case. Thus, in a hypothetical application of the device, it would be advised to slightly extend the range of the desired maximum magnetic field magnitude in productive mode for the training of the network.

The error Δ has a mean value of $\bar{\Delta }=23.8\;\mu T$ with a standard deviation of σ=47.7μT. We attribute this tendency of the CNN to predict slightly higher values than expected to a drift in the experimental setup in the time that has passed between training data acquisition and sensor performance testing. Possible sources of this drift can be of a thermal or mechanical nature, as well as laser power fluctuations. Single outliers in the measurement can be attributed to unsynchronized data acquisition due to randomly occurring timing errors in the control sequence put out by the ESP32, resulting in occasionally corrupted ODMR spectra.

To compare the performance of our method to a standard approach based on fitting a physical model to the data, an alternative method to extract the B-field magnitude from the presented ODMR spectra was used based on the theory of the NV ground state [15]. In the course of this, resonance frequencies were determined using a peak-finding algorithm [32] on the unfiltered, normalized data, described in detail in Appendix B. The total magnetic field magnitude can be estimated from these resonance frequencies. When applying this method to 30 of the acquired datasets, the resulting RMS is 10.4μT as compared to the value of 69.0μT achieved by the CNN. However, it should be noted that this method shows disadvantages compared to the CNN approach presented in this work. Most notably, while showing better sensitivity, the method can only be applied for spectra where all 8 resonances are clearly separated. This requirement ultimately limits the minimal detectable magnetic magnitude to 1.5mT in the case of the data presented in this work. Another important requirement is a good signal-to-noise ratio in the data acquisition system. A high-noise background can lead to error-prone peak detection, diminishing the reliability of the sensor. Furthermore, this method is not applicable in an environment where unknown external fields are present, as it only detects the total magnetic field magnitude.

#### 2.3.2. Repetition Rate of Magnetic Field Measurements

In our setup, the total measurement duration of the sensor device in productive mode is roughly 650ms, corresponding to a measurement rate of 1.54Hz. Per measurement cycle, performing inference takes only 608μs, which is a negligible contribution to the overall measurement time. Further, serial communication to transmit the predicted magnetic field magnitude only makes a small contribution to the measurement time in productive mode. Performing the frequency sweep and acquiring the ODMR spectrum amounts to 600ms and presents the largest limitation for the temporal resolution. In total, 400 steps are performed during the frequency sweep. The time needed to obtain a single MW frequency step (1.5ms) consists of the following main contributions. For each step, a software delay of 0.73ms is used to account for the SPI communication and 0.3ms for the settling of the phase-locked loop in the MW source. The ESP takes approx. 0.5ms per frequency step to obtain the ADC values and to average over the measurements of fluorescence intensity without any additional delays.

The main limitation for the temporal resolution of our sensor in productive mode is clearly the MW frequency sweep needed to acquire the ODMR spectra. Thus, the speed of the device can mainly be improved by using a faster sweeping microwave source or a faster ADC.

## 3. Conclusions and Outlook

In conclusion, we have presented a low-cost, stand-alone magnetometer sensor concept based on edge machine learning and ODMR in NV centers in diamond. In this proof-of-concept study, ODMR spectra were acquired in an automated manner by sweeping a microwave frequency while measuring the fluorescence intensity. Resulting spectra were used for training a convolutional neural network. The trained CNN was then flashed to the embedded device, where it performed inference on ODMR spectra acquired by the device’s internal ADC, predicting the external magnetic field magnitude applied. In our experiments, this prediction was accurate with a root-mean-square error of 69.0μT, which is comparable but not yet quite as precise as employing a conventional method on the obtained ODMR data, and overall is less precise than the sensitivity demonstrated in other publications [1,7,8,33,34]. We expect that in future work the precision can be further improved by using spectra with a larger number of data points, i.e., a finer resolved position of the peaks, by using smaller steps in setting the B field during training data acquisition or by using more complex and adapted network architectures with more and/or larger hidden layers. The systematic optimization of the network architecture by a large hyper-parameter study and optimization of the training process by employing techniques such as ensemble training was not within the scope of the present proof-of-concept study and will be performed in future work. Note that using a more complex network architecture would, in general, require a larger training set and potentially longer inference times. These could be compensated by employing quantization of the TFLite network, which reduces model size and speeds up computation. However, in our experiments, data acquisition was shown to have a far more substantial impact on the timing performance of the sensor in comparison to the inference times, making up approx. 93% of the total measurement time. The acquisition time is ultimately limited by the speed of the MW source and ADC. In this work, a phase-locked loop was used as a microwave source, which requires a set time to lock the MW frequency. Thus, the acquisition time can potentially be significantly improved by omitting the phase-locked loop and instead employing an open-loop VCO. While this would have a negative impact in a conventional ODMR magnetometer scheme, where closed-loop operation of the MW source is desired to extract the precise resonance frequencies, it should be very feasible with the presented machine learning approach.

Despite the room for improvement with respect to precision, the proposed measurement approach conceptually overcomes some of the challenges typically encountered in ODMR magnetometry. One advantage of the presented method is its robustness in the low B-field regime. Due to the superposition of the resonant transitions in the low magnetic field regime (Figure 4), it can be challenging to resolve single fluorescence dips in this regime and thus deduce the magnetic field magnitude from a measured ODMR spectrum with conventional methods based on the fit of a physical model. A common approach is to offset the magnetic field magnitude using a bias magnetic field [7,10]. However, with the presented machine learning approach, our experiments show no deterioration of precision in this low-field regime, omitting the need for a bias field. This can bring advantages in the application of magnetic sensing with NV ensembles, e.g., integrated sensor heads where a small form factor and a simplistic setup are desirable. Another conceptional advantage of the proposed approach is that it places only low requirements on the signal quality. Experiments have shown that the proposed sensor concept yields similar results for data with a worse signal-to-noise ratio than the data presented in this work, omitting the need for a balanced detection [1,35] or highly resolved data acquisition. Thus, we see potential for the sensor device to be applied in a non-laboratory environment with good reliability. The technical requirements to realize the proposed setup are low compared to other works in the field as the usage of lock-in amplifiers [2,34], modulated laser drivers and pulsed [2,36,37] or modulated [1,8] microwave excitation is not necessary.

Lastly, it is advantageous that with the presented approach, the alignment of the NV axes in micro-diamond, which proves to be challenging [9], can be neglected as long as the orientation of the magnetic field does not change with respect to the diamond over time. This point does not only apply to scalar magnetometry, but also to vector magnetometry. By labeling the acquired data with three-dimensional information (e.g., $B_{x}$, $B_{y}$, $B_{z}$), machine-learning-assisted vector magnetometry with randomly oriented micro-diamonds comes within reach and will be addressed in future works.

## Figures and Tables

**Table 1:** Comparison between the fully connected and the convolutional neural network in terms of the number of trainable parameters, the root-mean-square error on the test set after training and the size of the generated model.

Model	Number of Trainable Parameters	Root-Mean-Square Error on Test Set	Size of Generated Model
FCNN	6236	0.1392 mT	164 KB
CNN	5928	0.0636 mT	167 KB

**Figure A1:**
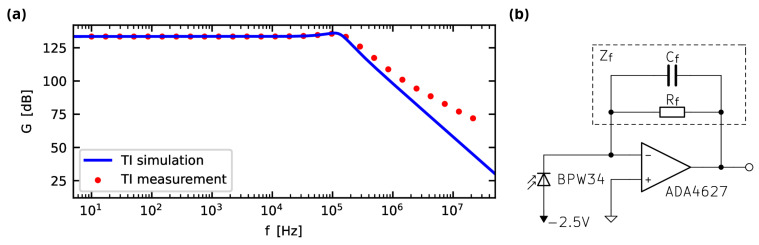
(**a**) Frequency response of the TIA for a gain of 4.7MΩ. (**b**) TI circuit. The photodiode was reverse-biased at −2.5V.

**Figure A2:**
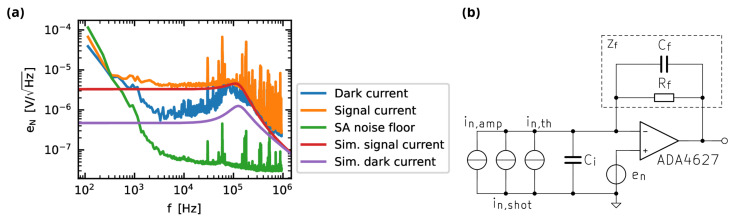
(**a**) Output noise spectral density of the TIA at signal current and dark current. The signal analyzer’s noise floor dominates below 1kHz. (**b**) Equivalent circuit showing individual noise current sources for the amplifier input current noise $i_{n,\mathrm{amp}}$, shot noise due to signal and dark current $i_{n,\mathrm{shot}}$ and thermal noise of $R_{f}$$i_{n,\mathrm{th}}$. The input voltage noise is modeled in the non-inverting input of the OPA. All parasitic input capacitances are included by $C_{i}$.

**Figure A3:**
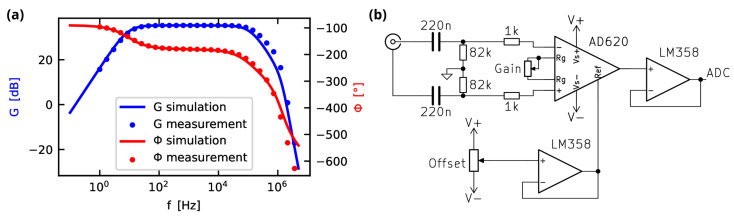
(**a**) Gain G and phase Φ of the analog processing circuit. Over a passband of 8Hz to 120kHz, the signal is inverted and amplified by 35dB. (**b**) The transfer function is implemented by a first-order high-pass filter followed by an Analog Devices AD620 instrumentation amplifier. Gain and offset are variable by trim potentiometers. The buffered output is fed into the ADC of the ESP32. Power is supplied by two 9 V batteries.

**Figure A4:**
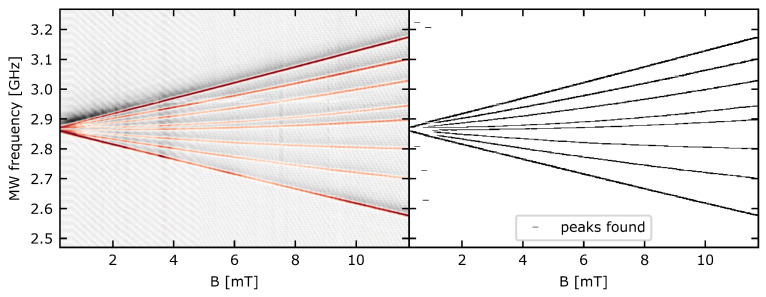
Peak finding in ODMR spectra in order to deduce the frequency values of the intensity dips. Note that for spectra below 1.5mT, peak finding is not reliable due to the superposition of multiple intensity dips.

## Data Availability

Data underlying the results presented in this paper are not publicly available at this time but may be obtained from the authors upon reasonable request.

## Abbreviations

The following abbreviations are used in this manuscript:

ODMR	Optically detected magnetic resonance
FCNN	Fully connected neural network
CNN	Convolutional neural network
CW	Continuous wave
MW	Microwave
NV	Nitrogen vacancy
ADC	Analog-to-digital converter
DAC	Digital-to-analog converter
TIA	Transimpedance amplifier

## Appendix A. Electronics

### Appendix A.1. Transimpedance Amplifier

To convert the fluorescence to a voltage signal, a transimpedance amplifier (TIA) was built, employing a BPW34 photodiode and an ADA4627 operational amplifier (OPA). The photodiode features a relatively large active area ($3\times 3\;{\mathrm{mm}}^{2}$) that eases the requirements on positional accuracy in the optical setup. At a reverse bias voltage of −2.5V its capacitance is reduced to under 30pF. In combination with a feedback resistance of $R_{f}=4.7\;M\Omega$, a bandwidth of 170kHz is achieved. To prevent oscillation, a feedback capacitor $C_{f}$ can be inserted. A simulation suggests a $C_{f}$ value of about 0.3pF for an open-loop phase margin of $45^{\circ }$, which was realized by parasitic capacitances only in this implementation. A comparison of simulation and measurement is shown in Figure A1a for the described circuit depicted in Figure A1b. The measured deviation for frequencies above 200kHz can be attributed to the test adapter’s parasitic capacitance in parallel to the resistance, which was used to feed a test current into the summing node. In the presented measurement setup, a DC output voltage of roughly 7V at zero magnetic field is given. The ratio of the feedback resistor’s thermal noise and shot noise by the signal current $i_{\mathrm{sig}}$ is given by

(A1) $$\frac{i_{n,\mathrm{th}}}{i_{n,\mathrm{shot}}}=\frac{\sqrt{4kT/R_{f}}}{\sqrt{2qi_{\mathrm{sig}}}}\approx 0.086$$

with Boltzmann’s constant *k*, elementary charge *q* and at room temperature T=300K. Thereby, the signal current shot noise dominates. To remain shot-noise-limited, the input current noise of the OPA has to stay well below $i_{n,\mathrm{shot}}\approx 0.69\;\mathrm{pA}/\sqrt{\mathrm{Hz}}$, leading to the choice of field-effect-based operational amplifiers. In a similar fashion, the OPA’s input voltage noise $e_{n,\mathrm{amp}}$ should stay below the feedback resistor’s thermal noise voltage $e_{n,\mathrm{th}}=\sqrt{4kTR_{f}}\approx 279\;\mathrm{nV}/\sqrt{\mathrm{Hz}}$, which is given with most OPAs. Furthermore, noise peaking due to $e_{n,\mathrm{amp}}$ in parallel to the input capacitance $C_{i}$ can be observed. For it not to dominate in the band of interest $f_{-3\mathrm{dB}}$, $e_{n,\mathrm{amp}}$ should also stay below $i_{n,\mathrm{th}}/\left(2\pi f_{-3\mathrm{dB}}\right)$ [38]. This requirement is also satisfied up to about 40kHz for the chosen OPA. Figure A2a shows the measured and simulated output noise spectral densities of the TIA at signal current and dark current. The 1/f-component of the signal analyzers used allows a meaningful measurement above 1kHz. For higher signal currents, good agreement can be achieved. At dark current only, the deviation of the measured noise can be attributed to the noisy environment. The noise sources can be modeled as shown in Figure A2a, resulting in an overall noise current

(A2) $$i_{n}=\sqrt{i_{n,\mathrm{amp}}^{2}+i_{n,\mathrm{shot}}^{2}+i_{n,\mathrm{th}}^{2}+{\left(s\;e_{n,\mathrm{amp}}\;C_{i}\right)}^{2}}.$$

The model’s output voltage noise density is then given by the transimpedance multiplied by the noise current. The 1/f-components of the OPA’s input current and voltage noise show no significant impact in the simulation down to 1Hz. At a signal output of 7V, an r.m.s. output noise of 2.78mV is given. The signal-to-noise ratio follows as 68dB.

### Appendix A.2. Inverting Buffer

Figure A3a shows the transfer function of the inverting buffer circuit, depicted in Figure A3b. It is used to preprocess the TIA signal for the ADC of the ESP32. The dips in fluorescence intensity ride on a large DC bias, which is high-pass filtered at a corner frequency of 8Hz. The signal is then inverted and amplified by an Analog Devices AD620 instrumentation amplifier. The gain is adjusted to make use of the ADC’s full-scale reference voltage. Due to the high-pass characteristic of the filter, peaks in the output signal are followed by a small undershoot. A positive offset is introduced to avoid clipping in these cases. A lower corner frequency of the high-pass filter would reduce this effect but has to be balanced with the rate of change of the DC bias, caused by the reduction in the fluorescence intensity through rising magnetic fields [39]. An LM358-based unity gain buffer passes the signal to the ADC. The circuit’s additional input noise density in the passband of $15\;\mathrm{nV}/\sqrt{\mathrm{Hz}}$ is insignificant in comparison to the TIA’s output noise density of $4.3\;\mu V/\sqrt{\mathrm{Hz}}$ at 7V DC, the output voltage given at zero magnetic field. The circuit’s output noise density then integrates to $96\;{\mathrm{mV}}_{\mathrm{rms}}$.

## Appendix B. Deduction of Magnetic Field Magnitude in ODMR Spectra with Automated Peak Finding

For a given ODMR spectrum, resonance frequencies are determined using a peak-finding algorithm [32] on the unfiltered and normalized data.

The total magnetic field magnitude is then calculated using

(A3) $$B_{tot}=\frac{\sqrt{3}}{2}\sqrt{\sum_{i=1}^{4}{\left(\frac{\Delta f_{i}}{2\gamma }\right)}^{2}},$$

where $\Delta f_{i}=f_{i,+}-f_{i,-},\;i\in \left\{1,2,3,4\right\}$ is the frequency difference in the Zeeman splitting for the $m_{S}=\pm 1$ spin states for all four NV orientations and γ=28 MHz/mT is the gyromagnetic ratio. Equation (A3) is based on the geometric properties of the NV axes in the diamond lattice. It follows from rearranging the expressions for the axial magnetic field for all 4 NV orientations $B_{i,\|}=\vec{B}\cdot {\vec{NV}}_{i}=\Delta f_{i}/\left(2\gamma \right)$ with the normalized vector representation of the four NV axes given by the Miller indices of the crystal lattice that describe the possible orientations [33] $\vec{N}V_{1}=\left(-1,-1,-1\right)/\sqrt{3},\;{\vec{NV}}_{2}=\left(-1,1,1\right)/\sqrt{3},\;{\vec{NV}}_{3}=\left(1,-1,1\right)/\sqrt{3}\;\mathrm{and}\;{\vec{NV}}_{4}=\left(1,1,-1\right)/\sqrt{3}.$
